# Enlarged adrenal glands: the long-term consequence of Cushing’s disease

**DOI:** 10.1007/s12020-019-01844-w

**Published:** 2019-02-04

**Authors:** Yorihiro Iwasaki, Akihiro Hamasaki

**Affiliations:** Center for Diabetes and Endocrinology, Tazuke Kofukai Medical Research Institute Kitano Hospital, 2-4-20 Ohgimachi, Kita-ku Osaka, 530-8480 Japan

Case report;

A 51-year-old woman presented with progressive swelling of the face with lower extremity edema. At age 38, she was initially diagnosed as having Cushing’s disease caused by a pituitary corticotroph adenoma (Fig. 1a). At that time, bilateral adrenal glands were not enlarged (Fig. 1b). Initially, she underwent transsphenoidal surgery. Pathological diagnosis was sparsely granulated corticotroph adenoma with positive immunostaining for adrenocorticotropic hormone (ACTH). Ki-67-positive cells were less than 1%. Postoperatively, however, residual tumor remained in the cavernous sinus. Postoperative pituitary irradiation was ineffective to control ACTH. Octreotide and cabergoline were also ineffective, and pasireotide had not been approved for Cushing’s disease in Japan. Her plasma ACTH remained high, around 100 pg/ml (normal range 7.2–63.3 pg/ml), and thereafter, her hypercortisolemia had been medically managed with metyrapone, a steroidogenesis inhibitor, over 10 years (Fig. 1a). She could not tolerate mitotane, an adrenolytic agent, because of the adverse effect of gastrointestinal disturbances. At age 51, bilateral adrenal glands became greatly enlarged (Fig. 1c), and hypercortisolemia became uncontrollable with high-dose metyrapone (4000–6250 mg/day, Fig. 1a). Consequently, systemic edema, hyperglycemia, hypertension, and hypokalemia worsened progressively. Therefore, she underwent laparoscopic bilateral adrenalectomy (Fig. 1d). Pathologically, enlarged bilateral adrenal glands were composed of zona fasciculata-like cells with no evidence of mitotic figures, necrosis, or vascular invasions. Following adrenalectomy, she was on hydrocortisone replacement therapy. Twelve months after adrenalectomy, she remains well, and there is no sign of remaining pituitary tumor growth on magnetic resonance imaging (Fig. 1a).

**Fig. 1:**
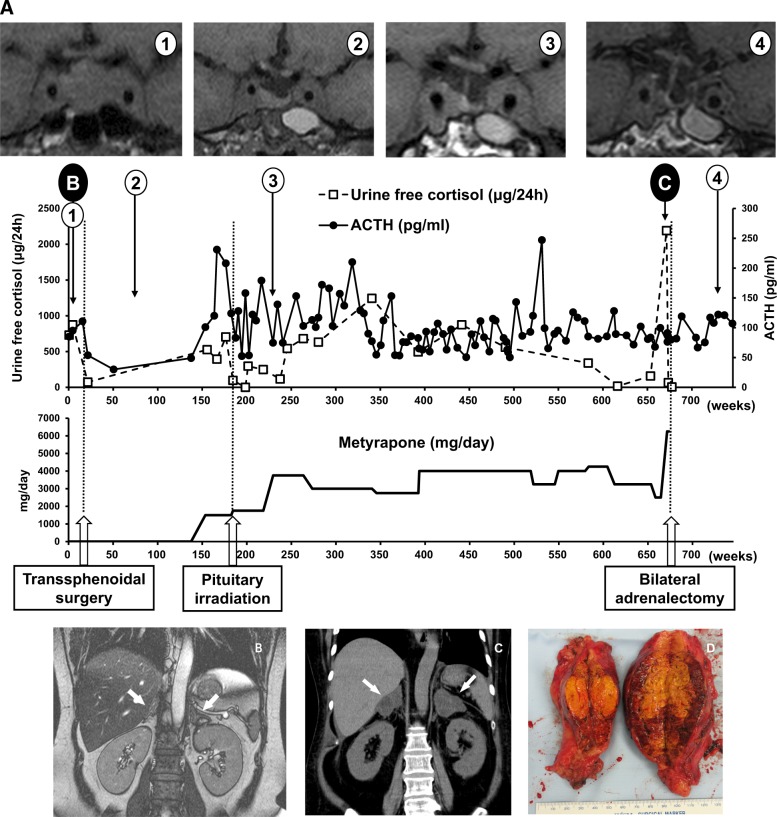
**a** The time course of treatment. Horizontal axes of the upper and lower graphs show weeks from the time of diagnosis. In the upper graph, changes in urine free cortisol in 24 h urine specimens (dashed line) and plasma ACTH (solid line) are shown. In the lower graph, changes in metyrapone dose are shown. At the bottom, the timings of transsphenoidal surgery, pituitary irradiation, and bilateral adrenalectomy are shown with arrows and dashed lines. Four pituitary T1-weighted coronal MRI images on the top were taken at the indicated time points (marked as “1”–“4”). Black-circled “B” and “C” are the time points when **b** and **c** imaging studies were performed, respectively. **b** Abdominal MRI of bilateral adrenal glands (arrows) at the time of diagnosis of Cushing’s disease. **c** Abdominal CT showing enlarged bilateral adrenal glands (arrows) 13 years after diagnosis of Cushing’s disease. **d** Surgically resected right (left on the photograph) and left (right on the photograph) adrenal glands

In patients with Cushing’s disease who are refractory to initial treatments for pituitary tumor, hypercortisolemia must be managed medically or surgically [1]. Although bilateral adrenalectomy is the most reliable option for the treatment of hypercortisolemia, it may be associated with potential risk of pituitary tumor growth, known as Nelson’s syndrome [2]. Therefore, physicians are often reluctant to decide adrenalectomy [3]. On the other hand, the “escape” phenomenon from response has been reported in the long-term treatment with steroidogenesis inhibitors [1]. Although the underlying mechanism of the phenomenon remains unclear, as for metyrapone, long-term treatment may result in “escape” in 4–13% of patients (Table 1) [4–6]. A plausible explanation of “escape” in the current case would be that long-term stimulation by ACTH lead to marked enlargement of adrenal glands with robust production of cortisol, which could not be suppressed by high-dose metyrapone. Another possibility is that enlarged adrenal glands autonomously secreted cortisol, as suggested by previous reports [1, 7, 8]. However, because of persistently elevated plasma ACTH in the current case, it is difficult to determine whether cortisol was autonomously produced independently of ACTH.

**Table 1:** Review of the literature of the “escape” phenomenon in long-term metyrapone-treated patients with Cushing’s syndrome

Authors	Number of cases	Number of “escape”	Duration of treatment	Morphological changes in adrenal glands	Reference
Verhelst et al.	24	1	3–140 months	Not characterized.	[4]
Valassi et al.	23	3	1–30.7 months	Not characterized.	[5]
Ceccato et al.	31	3	3–12 months (interquartile range)	Not characterized.	[6]

In conclusion, the current case highlights the difficulty in long-term treatment of Cushing’s disease, especially with metyrapone. The optimal timing for bilateral adrenalectomy is not clearly defined, and physicians must make difficult decisions in the management of refractory Cushing’s disease.

## References

[1] Pivonello R, De Leo M, Cozzolino A, Colao A (2015). The treatment of Cushing’s disease. Endocr. Rev..

[2] Nelson DH, Meakin JW, Dealy JB, Matson DD, Emerson K, Thorn GW (1958). ACTH-producing tumor of the pituitary gland. N. Engl. J. Med..

[3] Morris LF, Harris RS, Milton DR, Waguespack SG, Habra MA, Jimenez C, Vassilopoulou-Sellin R, Lee JE, Perrier ND, Grubbs EG (2013). Impact and timing of bilateral adrenalectomy for refractory adrenocorticotropic hormone-dependent Cushing’s syndrome. Surgery.

[4] Verhelst JA, Trainer PJ, Howlett TA, Perry L, Rees LH, Grossman AB, Wass JA, Besser GM (1991). Short and long-term responses to metyrapone in the medical management of 91 patients with Cushing’s syndrome. Clin. Endocrinol. (Oxf.)..

[5] Valassi E, Crespo I, Gich I, Rodríguez J, Webb SM (2012). A reappraisal of the medical therapy with steroidogenesis inhibitors in Cushing’s syndrome. Clin. Endocrinol. (Oxf.)..

[6] Ceccato F, Zilio M, Barbot M, Albiger N, Antonelli G, Plebani M, Watutantrige-Fernando S, Sabbadin C, Boscaro M, Scaroni C (2018). Metyrapone treatment in Cushing’s syndrome: a real-life study. Endocrine.

[7] Pereira AM, van Aken MO, van Dulken H, Schutte PJ, Biermasz NR, Smit JW, Roelfsema F, Romijn JA (2003). Long-term predictive value of postsurgical cortisol concentrations for cure and risk of recurrence in Cushing’s disease. J. Clin. Endocrinol. Metab..

[8] Valassi E, Biller BM, Swearingen B, Pecori Giraldi F, Losa M, Mortini P, Hayden D, Cavagnini F, Klibanski A (2010). Delayed remission after transsphenoidal surgery in patients with Cushing’s disease. J. Clin. Endocrinol. Metab..

